# Antimicrobial Stewardship Intervention and Feedback to Infectious Disease Specialists: A Case Study in High-Dose Daptomycin

**DOI:** 10.3390/antibiotics4030309

**Published:** 2015-07-24

**Authors:** Jennifer L. Ross, Shannon Rankin, Patricia Marshik, Renée-Claude Mercier, Meghan Brett, Carla J. Walraven

**Affiliations:** 1 Department of Pharmaceutical Services, University of New Mexico Hospital, 2211 Lomas Blvd NE, Albuquerque, NM 87106, USA; E-Mails: jeross@salud.unm.edu (J.L.R.); srankin@salud.unm.edu (S.R.); 2 College of Pharmacy, University of New Mexico Health Sciences Center, MSC09 5360, 1 University of New Mexico, Albuquerque, NM 87131, USA; E-Mails: pmarshik@salud.unm.edu (P.M.); rmercier@salud.unm.edu (R.-C.M.); 3 Division of Infectious Diseases, Department of Internal Medicine, 1 University of New Mexico, Albuquerque, NM 87131, USA; E-Mail: mbrett@salud.unm.edu

**Keywords:** antimicrobial stewardship, daptomycin, infectious diseases

## Abstract

Infectious Diseases specialists have used high-dose daptomycin (≥6 mg/kg/day) in select patients with difficult to treat methicillin-resistant *Staphylococcus aureus* (MRSA) or vancomycin-resistant Enterococcus (VRE) infections to optimize outcomes. Antimicrobial stewardship programs enforce antimicrobial formulary restrictions; however, interventions specifically aimed at Infectious Disease specialists can be particularly challenging. The purpose of this study was to create a high-dose daptomycin algorithm for Infectious Disease specialists that are consistent with best-practices. Daptomycin prescribing habits pre- and post-daptomycin algorithm implementation were evaluated using a quasi-experimental study design. Patients were included if ≥18 years of age and received daptomycin for ≥48 h. Patients were excluded if daptomycin was initiated on an outpatient setting. During the 12-month pre-intervention phase, 112 patients were included, with 73 patients in the 12-month post-intervention phase. A statistically significant decrease in the mean daptomycin dose from 9.01 mg/kg to 7.51 mg/kg (*p* < 0.005) was observed, resulting in an annual drug cost-savings of over $75,000 without adversely affecting readmission rates due to infection. Creation of a daptomycin algorithm with consideration of pathogen, disease state, and prior treatment, is an effective means of influencing prescribing habits of Infectious Disease specialists.

## 1. Introduction

Daptomycin is a novel lipopeptide antibiotic that has activity against a variety of Gram positive pathogens, including methicillin-resistant *Staphylococcus aureus* (MRSA), heteroresistant vancomycin-intermediate *S. aureus* (hVISA) and *Enterococcus* spp., including vancomycin-resistant Enterococci (VRE) [1,2]. Daptomycin is approved by the United States Food and Drug Administration (FDA) at 4 mg/kg for the treatment of complicated skin and soft tissue infections (SSTI) caused by Gram positive bacteria and 6 mg/kg for *S.aureus* bacteremia, including those associated with right-sided endocarditis [3]. In clinical practice, daptomycin is reserved for patients who have failed, or are intolerant to vancomycin therapy. In disease states such as endocarditis and osteomyelitis, which often require prolonged durations of therapy, daptomycin is used since it is dosed once-daily, does not cause nephrotoxicity, and does not require drug concentration monitoring for safety or efficacy.

Daptomycin has linear concentration-dependent bactericidal activity, meaning higher doses are anticipated to optimize clinical efficacy [4]. In some of the early studies of daptomycin for Staphylococcal infections, standard daptomycin dosing (6 mg/kg) was associated with inferior microbiological eradication and reduced clinical cures compared to high-dose therapy (>6 mg/kg) [5,6]. *In vivo* and *in vitro* studies of clinical *S. aureus* strains have described a phenomenon in which prior exposure to vancomycin resulted in reduced daptomycin susceptibilities [7,8]. This phenomenon was documented in a case report by Bennett *et al.* [9], of a 94-year old woman with persistent MRSA bacteremia despite optimization of vancomycin to obtain serum levels >15 µg/mL. After 15 days of vancomycin therapy, daptomycin was initiated at 6 mg/kg/day. However repeat blood culture results indicated that the daptomycin minimum inhibitory concentration (MIC) had increased from 0.75 µg/mL at baseline to 4 µg/mL. The patient’s daptomycin was increased to 8 mg/kg/day to overcome the elevated MICs but the patient ultimately had a poor outcome [9]. 

The mechanisms by which daptomycin becomes non-susceptible are not fully understood, but are believed to be multi-factorial [10]. Disease states associated with a high inoculum, such as endocarditis, may contribute to attenuated daptomycin activity where the susceptible organisms are killed, leaving a sub-population of less susceptible organisms. In addition, *S. aureus* isolates with MICs of 2 µg/mL are more likely to have higher frequencies of hVISA, which may also attenuate daptomycin activity. Finally, structural mutations that increase the thickness of the cell wall, thereby impairing the ability of daptomycin to reach its target may also contribute to attenuated daptomycin activity [11,12]. Infectious Disease experts have advocated for high-dose daptomycin in select patients with persistent MRSA bacteremia/endocarditis or systemic VRE infections that have previously been exposed to vancomycin to optimize clinical outcomes with daptomycin [13,14,15,16,17]. Falcone and colleagues evaluated the clinical outcomes of critically ill patients treated with high-dose daptomycin (6–8 mg/kg) and found that septic patients with MRSA bacteremia/endocarditis had higher rates of daptomycin clearance and were significantly associated with worse clinical outcomes, possibly due to suboptimal drug exposures. However, they could not find a strong correlation between daptomycin clearance and body weight that would allow one to identify this subset of patients. Through pharmacokinetic modeling, they confirmed that higher daptomycin doses (8–10 mg/kg) would achieve the desired target of attainment; however it was also more likely to result in higher trough concentrations, which has been associated with skeletal muscle toxicity. They proposed a fixed daptomycin dosing scheme of 500 mg for non-septic patients and 750 mg for septic patients to achieve the desired target of attainment while minimizing skeletal muscle toxicity rather than a weight-based dosing scheme [18]. Other than a case report of high-dose daptomycin (8 mg/kg) to treat a persistent *Staphylococcus epidermidis* transjugular intrahepatic portosystemic shunt (TIPS) infection, there is limited data to support its use for non-MRSA and non-VRE Gram positive infections [19,20].

At the University of New Mexico Hospital (UNMH), a 646-bed, tertiary care, academic level I trauma center in Albuquerque, New Mexico, there is an active Antimicrobial Stewardship program that reviews broad spectrum and restricted antibiotics on a daily basis. Daptomycin initiation is restricted to Infectious Disease specialists and is prescribed at their discretion with no further Antimicrobial Stewardship interventions. In 2013, daptomycin accounted for one-quarter of all antimicrobial drug costs combined. Upon further review, a comparison of daptomycin utilization from 27 academic medical centers in the University HealthSystem Consortium (UHC) database with ≥500 beds from 2012 to 2013 indicated the average daptomycin dose was 638 mg (range 475 mg to 772 mg), or 7.5 mg/kg/dose when standardized to an 85-kg patient. In contrast, the average daptomycin dose at UNMH was 1.5 times the benchmark daptomycin dose during the same time frame. This discrepancy in practice could not be attributed to an increase in antibiotic resistance based on local antibiogram trends. Furthermore, UNMH lacks many of the service lines, such as inpatient rehabilitation, a hospice unit, and long term care facilities, which are often associated with more antibiotic resistant infections.

Therefore, the purpose of this study was to create a high-dose daptomycin dosing algorithm with education of Infectious Disease specialists to reduce excessive daptomycin prescribing practices at our institution. The secondary aims were to describe adverse events associated with high-dose daptomycin, readmission rates, and costs associated with the use of high-dose daptomycin prescribing habits.

## 2. Methods

### 2.1. Study Timeline

This study consisted of three phases: (1) a baseline audit of daptomycin prescribing habits at UNMH from 1 October 2012 through 30 September 2013; (2) development of a daptomycin dosing algorithm, hospital approval, and education of Infectious Disease specialists from 1 October 2013 to 31 December 2013; (3) a post-intervention audit of daptomycin prescribing habits from 1 January 2014 to 31 December 2014. This data was then compared to the baseline data with subsequent feedback of results to Infectious Disease specialists. This study was approved by the University of New Mexico Human Research Review Committee.

### 2.2. Data Collection

Patients were included in this study if they were ≥18 years of age at the time of their hospital admission and received daptomycin for ≥48 h between 1 July 2012 and 31 December 2014. Patients were excluded if daptomycin was initiated in the outpatient setting. Data was retrospectively collected from patients’ electronic medical charts using a standardized data collection form. Data collected included patient age, gender, race, height, weight, antibiotic allergies, renal function, and creatine phosphokinase (CPK), both at baseline and during therapy. Microbiological culture results including site(s), pathogen, and antibiotic susceptibilities were recorded. All microbiological susceptibilities were performed at the reference laboratory using the BD Phoenix automated system. Antibiotic related data elements included recent antibiotic(s) administered during hospitalization, daptomycin dose (with mg/kg calculated based upon the total body weight documented on the day of daptomycin initiation), frequency, duration, and indication for daptomycin initiation. Clinical outcomes including daptomycin-related adverse drug events and infection-related readmissions occurring within 30-days of discharge were recorded for each patient.

### 2.3. Cost-Analysis

At our institution, daptomycin is not batched or rounded to the nearest vial to conserve costs. A cost-analysis was calculated based on the difference in the mean daptomycin dose (in mg/kg) for a standard 85-kg patient in the pre- and post-intervention phases. Therefore, to estimate cost savings, the drug acquisition cost per milligram of drug ($0.67/mg) was multiplied by an annualized average number of daptomycin doses. The average number of daptomycin doses per year was calculated by factoring in the mean duration of therapy per patient while hospitalized.

### 2.4. Daptomycin Dosing Algorithm

A daptomycin dosing algorithm was developed in conjunction with Infectious Disease specialists, based on the suspected or confirmed pathogen and disease state with considerations for FDA-approved dosing recommendations, Infectious Diseases Society of America MRSA treatment recommendations and evidence-based reports of high-dose daptomycin use (Table 1) [18,20,21,22]. Pneumonia and urinary tract infections were excluded and dosing was based on the total body weight of the patient at the time of daptomycin initiation. This algorithm was approved by the Antimicrobial Subcommittee and Pharmacy and Therapeutics committees. Education regarding the utilization of the algorithm was provided to Infectious Disease specialists, including infectious disease fellows and mid-level providers during their weekly conference. 

### 2.5. Statistical Analysis

Descriptive statistics were used to describe demographic data in the pre- and post-implementation phases. Daptomycin dosing pre- and post-intervention was analyzed using an independent *t*-test. A Pearson correlation was used to determine if a correlation existed between body mass index (BMI) and peak CK levels. *p*-Values ≤ 0.05 were considered statistically significant.

**Table 1:** Daptomycin dosing algorithm.

Pathogen	SSTI	Severe Infection or Difficult to Treat ^a,b^
Coagulase negative Staphylococci (CoNS)	4–6 mg/kg IV daily	6 mg/kg IV daily
MSSA	4–6 mg/kg IV daily	6–8 mg/kg IV daily
MRSA	4–6 mg/kg IV daily	8–10 mg/kg IV daily
*Enterococcus* spp. ^c^	4–6 mg/kg IV daily	6 mg/kg IV daily
VRE	4–6 mg/kg IV daily	8–10 mg/kg IV daily

^a^ Severe Infection includes bacteremia, endocarditis, osteomyelitis or septic arthritis; ^b^ Difficult to treat infections are those that have failed to respond to appropriate antimicrobial therapy or recurrence of infection while on appropriate antimicrobial therapy; ^c^ For penicillin allergic patients.

## 3. Results and Discussion

During the 12-month pre-intervention phase, there were 112 adult patients who met the inclusion criteria. There were 73 patients who met the inclusion criteria during the 12-month post-implementation phase. Baseline demographics were consistent between both study phases. Comorbid conditions were not statistically different between the study phases (Table 2). The mean documented patient weight was 85.1 kg in the pre-intervention and 86.3 kg in the post-intervention phase. Allergies to vancomycin were documented in 14 of the 112 patients pre-intervention and in 13 of 73 patients post-intervention. A penicillin allergy was documented in 15 of 112 patients pre-intervention and in 10 of 73 patients in the post-intervention.

Daptomycin was used in lieu of vancomycin in 53% of patients in the pre-intervention and in 53% of patients post-intervention. Similarly, daptomycin replaced cephalosporins in 10% of patients pre-intervention and 6.8% of patients post-intervention; nafcillin in 10% of patients pre-intervention and 4.1% of patients post-intervention; linezolid in 7.1% of patients pre-intervention and 13.7% of patient post-intervention. Reasons commonly cited for daptomycin initiation included: culture results in 29.5% of patients pre-intervention and 16.4% of patients post-intervention; acute kidney injury (AKI) from the previous antibiotic in 16.1% of patients pre-intervention and 19.1% of patients post-intervention; adverse drug event other than AKI in 8% of patients pre-intervention and 17.8% of patients post-intervention; convenience for outpatient drug administration in 11% of patients pre-intervention and 13.7% of patients post-intervention; and a desire to broaden coverage in 8.9% of patients pre-intervention and 16.4% of patients post-intervention.

Daptomycin was empirically initiated in 24.1% of patient in the pre-intervention phase and in 50.7% of patients in the post-implementation phase by Infectious Disease specialists. Daptomycin was most commonly prescribed for treatment of osteomyelitis (OM)/septic joint infections in 37% of patients pre-intervention *vs.* 26% of patients post-intervention; bacteremia in 31% of patients pre-intervention *vs.* 32% of patients post-intervention; and SSTI in 19% of patients pre-intervention *vs.* 26% of patients post intervention (Table 3).

**Table 2:** Patient Demographics.

Characteristic	Pre-Intervention (*n* = 112)	Post-Intervention (*n* = 73)	*p*-value
Mean age (range), years	54 (20–87)	55 (21–83)	0.544
Gender, no. (%)			0.178
Male	68 (60.7)	37 (50.7)	
Female	44 (39.3)	36 (49.3)	
Race/ethnicity, no. (%)			
White	52 (46.4)	47 (64.4)	0.016
Hispanic	28 (25.0)	0	<4 × 10^−6^
American Indian	14 (12.5)	10 (13.7)	0.812
Other	7 (6.3)	0	0.029
Unknown	11 (9.8)	16 (21.9)	0.649
Body Mass Index, kg/m^2^			
<25	32 (28.6)	15 (20.5)	0.220
25–29.9	29 (25.9)	23 (31.5)	0.406
30–39.9	41 (36.6)	27 (37.0)	0.958
≥40	10 (8.9)	8 (11.0)	0.649

**Table 3:** Infection site and isolated pathogens in patients started on daptomycin.

Variable	Pre-Intervention	Post-Intervention	*p*-value
	*n* (%)	*n* (%)	
Infection site ^a^			
OM/septic arthritis	54 (36.7)	23 (25.6)	0.024
Bacteremia	46 (31.3)	29 (32.2)	0.855
SSTI	28 (19.0)	23 (25.6)	0.333
Endocarditis	5 (3.4)	4 (4.4)	0.754
Abdominal	7 (4.8)	4 (4.4)	0.829
Isolated Gram positive pathogen(s)			
MRSA	29 (23.6)	10 (13.7)	0.047
VRE	29 (23.6)	16 (21.9)	0.538
MSSA	16 (13.0)	7 (9.6)	0.344
*E. faecalis*	13 (10.6)	7 (9.6)	0.666
CoNS	8 (6.5)	2 (2.7)	0.195
*S. pneumoniae*	1 (0.8)	0	0.418
Other *Streptococcus* spp. ^b^	5 (4.1)	2 (2.7)	0.548

^a^ Skin and soft tissue infections encompassed cellulitis, wounds, abscesses, automatic implantable cardioverter defibrillator (AICD) pockets, pericardial tissue, and vulva. Abdominal infections included pathogens identified from the peritoneal fluid, pancreatic fluid, or bile; ^b^ Other *Streptococcus* spp. includes: *S. intermedius*, *S. gordonii*, *S. anginosus*, and beta-hemolytic streptococcus.

Isolated Gram positive pathogens are listed in Table 3, with the following pathogens identified in more than 10% of patients: MRSA, VRE, methicillin-sensitive Staphylococcus aureus (MSSA), and ampicillin-sensitive *E. faecalis*. There was one patient (0.9%) in the pre-implementation phase with a MRSA isolate with a vancomycin MIC of 2 µg/mL and one patient (1.4%) with a vancomycin MIC of 2 µg/mL in the post-implementation phase. There were no patients in either phase with a daptomycin non-susceptible isolate. Polymicrobial infections were documented in 5% of patients pre-intervention and 3% of patients post-intervention. There were 20 (16.3%) patients pre-intervention and 21 (28.8%) patients post-intervention who did not have a Gram positive pathogen isolated from cultures.

**Figure 1:**
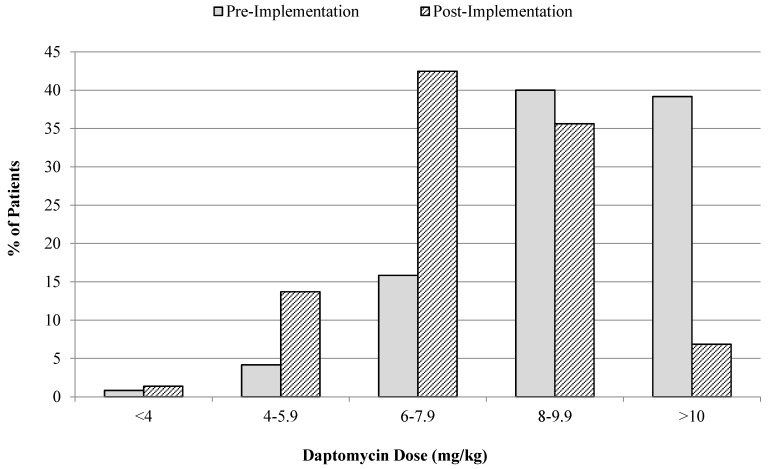
Mean daptomycin dose pre- and post-intervention. Doses are based on the total body weight documented at the time of daptomycin initiation.

In the pre-intervention phase of the study, the mean daptomycin dose was 9.01 ± 1.75 mg/kg, with 80.4% of patients receiving doses in excess of 8 mg/kg. After implementation of the daptomycin dosing algorithm, there was a statistically significant decrease in the mean daptomycin dose to 7.51 ± 1.56 mg/kg (*p* < 0.005), with 42.5% of patients receiving doses in excess of 8 mg/kg. The majority of patients (78.1%) in the post-intervention phase received doses between 6 mg/kg and 9.9 mg/kg (Figure 1). A significant decrease in the percentage of patients receiving daptomycin doses ≥10 mg/kg was noted after the intervention (39.2% *vs.* 6.7%). The maximum observed dose was 14.3 mg/kg in the pre-intervention and 10.4 mg/kg in the post-intervention phase. The daptomycin dosing decreased from the pre-intervention to the post-intervention phases for all types of infections, but was most notable for SSTIs where the mean dosing was 8.96 mg/kg pre-intervention compared to 6.64 mg/kg in the post-intervention phase. For infections where MRSA or VRE were isolated, there was a statistically significant decrease in the mean daptomycin dosing from 9.15 and 9.17 mg/kg, respectively in the pre-intervention phase to 7.32 and 7.88 mg/kg, respectively in the post-intervention phase (*p* = 0.008 and *p* = 0.024 respectively). There was a significant decrease in the mean daptomycin dosing observed for infections involving ampicillin susceptible *Enterococcus* spp. from 9.43 mg/kg in the pre-intervention phase to 7.37 mg/kg in the post-intervention phase (*p* = 0.065), which is still higher than the recommendations outlined in the daptomycin dosing algorithm.

The mean inpatient duration of therapy was similar between the two groups: 10.7 days in the pre-intervention and 10.4 days in the post-intervention phase (*p* = 0.440). Adverse drug events leading to the discontinuation of daptomycin were noted in 6.3% of patients pre-intervention and 1.4% of patients post-intervention. There was one instance of eosinophilic pneumonia, with all other adverse events related to CK elevations. There was no statistically significant correlation observed between BMI and CK elevation in either the pre-implementation or post-implementation phases (*p* = 0.521 and *p* = 0.530), respectively. There were 8% of patients pre-intervention and 5.5% of patients post-intervention with infection-related readmissions within 30-days of discharge from their original hospitalization, which was not statistically significant (*p* = 0.688).

### 3.1. Cost-Analysis

A cost-analysis was calculated using the difference between the mean daptomycin dose pre- and post-implementation. For a patient weighing 85-kg, the pre-intervention dose would be 765 mg, compared to 640 mg post-intervention, resulting in a difference of 125 mg of daptomycin, or $84.06 using the drug acquisition cost of $0.67/mg of daptomycin. Using a mean inpatient duration of daptomycin therapy of 10 days, this yields an average of 909 doses of daptomycin per year, leading to an annual drug acquisition cost-savings of $76,412.

Creation of a daptomycin dosing algorithm aided Infectious Disease specialists in selecting patients who would be most likely to benefit from high-dose daptomycin therapy, based on culture results, prior treatment history, and disease state. The mean post-intervention daptomycin dose decreased to 7.5 mg/kg, which is more consistent with prescribing habits seen at other academic medical institutions.

To our knowledge, Tran and colleagues are the only other group to implement an Antimicrobial Stewardship driven high-dose daptomycin protocol to optimize dosing for VRE infections in which few treatment options exist [17]. In our study, high-dose daptomycin (8–10 mg/kg) was the predominant dosing regimen for all infections, regardless of prior treatment history, pathogen, or the disease state.

A notable finding from this study was that about half of the patients on daptomycin in each phase were obese or morbidly obese with BMIs ≥30 kg/m^2^. Increased patient weight has been associated with larger vancomycin doses and potentially an increased risk of AKI when optimized to maintain troughs >15 µg/mL [23,24]. Approximately 20% of patients in each study phase were switched to daptomycin as a result of AKI with their previous antibiotic, usually vancomycin. Since daptomycin is frequently dosed upon total body weight, regardless of BMI, this can substantially increase drug costs [25]. Recent literature suggests that obese patients may achieve similar outcomes utilizing ideal or adjusted body weight for dosing, rather than total body weight, without an increase in the length of hospitalization, mortality or adverse events [26,27,28]. Other investigators have noted a potential correlation between increased BMI and CK elevations when dosed based upon total body weight, possibly due to higher serum levels [29,30]. Although a similar correlation was not observed in this study, daptomycin use in obese or morbidly obese patients warrants further investigation about which dosing weight can be safely used to optimize patient outcomes. Many new alternative antibiotics, such as ceftaroline, dalbavancin, and linezolid or tedizolid, may offer an alternative to daptomycin for obese patients which are not dependent on the patient’s weight.

There are several limitations to our study that should be recognized. First, this was a retrospective, dosing study, with observed decreases in daptomycin doses attributable solely to educational efforts. In the post-intervention phase, there was a decrease in the number of daptomycin doses prescribed, however this did not correspond to increased prescribing of linezolid, which is the formulary alternative to daptomycin and vancomycin. There were significantly less infections with osteomyelitis and septic arthritis in the post-intervention phase which may have contributed to the decrease in daptomycin use; however, high-dose daptomycin (>6 mg/kg) was still utilized for all types of infections and when MRSA, VRE, MSSA, and *Enterococcus* spp. were isolated. While the pre- and post-implementation time frames encompass the same duration, differences in seasonality and the types of infections necessitating Gram positive coverage may have influenced prescribing habits for daptomycin. When the results of this study were presented to the Infectious Disease specialists, many misconceptions were dispelled, including the need to round to the nearest vial size and that the standard daptomycin dose was 10 mg/kg instead of 4–6 mg/kg. Last, our daptomycin dosing was overly aggressive compared to other academic institutions and therefore, may not be generalizable to institutions with less aggressive dosing habits.

Many Antimicrobial Stewardship initiatives are focused on improving antimicrobial prescribing to reduce inappropriate antimicrobial use. In addition to more passive measures that target inappropriate antimicrobial use, such as hospital protocols, automatic substitutions, and formulary restrictions, Antimicrobial Stewardship programs have also targeted specific providers, (e.g., Emergency Department, Intensive Care Units, hospitalists) in an effort to re-align inappropriate prescribing patterns with best practices. In areas where Infectious Disease specialists are available, they often serve as an invaluable support for Antimicrobial Stewardship interventions. While Infectious Disease specialists are trained to make the most appropriate antimicrobial recommendations, and would seem like natural antimicrobial stewards, their recommendations are not necessarily consistent with the goals of Antimicrobial Stewardship programs. This point was demonstrated at the University of Maryland Medical Center in which the duties of their Antimicrobial Stewardship program were felt to be redundant to their Infectious Disease specialists and was therefore dismantled. A continued evaluation of their hospitals’ length of stay, readmissions, and mortality demonstrated no change, yet there was an increase in antimicrobial costs without any clear benefits [31]. Despite the overlapping interests of Infectious Disease specialists and Antimicrobial Stewardship programs, the day-to-day goals and responsibilities differ between the two groups and cannot be assured or assumed by the other group. Therefore, Antimicrobial Stewardship programs may still need to intervene with Infectious Disease specialists when an identified prescribing habit falls outside of the norm.

## 4. Conclusions

This study demonstrates that Antimicrobial Stewardship programs can have an impact on antimicrobial prescribing habits of Infectious Disease specialists. By working with Infectious Disease specialists, we were effectively able to design and implement a high-dose daptomycin dosing algorithm that incorporates pathogen, disease state, and prior treatment history that is in line with best clinical practices while minimizing excessive healthcare costs.
